# Sex Difference in Female and Male Ice Swimmers for Different Strokes and Water Categories Over Short and Middle Distances: A Descriptive Study

**DOI:** 10.1186/s40798-022-00451-w

**Published:** 2022-05-10

**Authors:** Janne Oppermann, Beat Knechtle, Aldo Seffrin, Rodrigo Luiz Vancini, Claudio Andre Barbosa de Lira, Lee Hill, Marilia Santos Andrade

**Affiliations:** 1 Institute of Primary Care, University Hospital Zurich, Zurich, Switzerland; 2 Medbase St. Gallen Am Vadianplatz, Vadianstrasse 26, 9001 St. Gallen, Switzerland; 3 Department of Physiology, Federal University of São Paulo, São Paulo, Brazil; 4 Center for Physical Education and Sports, Federal University of Espírito Santo, Vitoria, Brazil; 5 Human and Exercise Physiology Division, Faculty of Physical Education and Dance, Federal University of Goiás, Goiânia, Brazil; 6 Division of Gastroenterology and Nutrition, Department of Pediatrics, McMaster University, Hamilton, Canada

**Keywords:** Aquatic sports, Cold, Sex, Sport, Swimming, Water temperature

## Abstract

**Background:**

Winter swimming developed from a national tradition into a health-improving sport with international competitions. The difference in performance between women and men was thoroughly examined in various sporting disciplines; however, there is little data on winter swimming events. Therefore, this study aims to compare the sex differences in female and male winter swimmers for a distinct stroke over distances of 25 m and 200 m in ice water, freezing water and cold water in the multiple stages of the Winter Swimming World Cup, hosted by the International Winter Swimming Association (IWSA) since 2016.

**Methods:**

All data included in this study were obtained from the official results of the Winter Swimming World Cup, published on the “International Winter Swimming Association” (IWSA) website. The Mann–Whitney *U* test was used to compare race time between sexes in different swimming strokes and categories of water. In contrast, the Kruskal–Wallis *H* test was used to compare differences between swimming strokes or water categories for the same sex.

**Results:**

For 25 m and 200 m events of the “IWSA World Cup,” male athletes were faster than female athletes, regardless of stroke and water temperature category. However, the effect size of the difference between the sexes was greater in 25 m than in 200 m for all strokes and water temperatures. Swimming speed for the same-sex differed between the swimming stroke in relation to the water temperature category. Head-up breaststroke was found to be the slowest stroke (*p* < 0.05).

**Conclusion:**

In water temperatures between − 2° and + 9 °C, men were faster than women in all stages of the “IWSA World Cup,” regardless of the swimming stroke, but the effect size of the difference between the sexes was greater in shorter than in longer events.

## Key Points

- Males are faster than females in ice swimming, most likely due to a higher muscle mass and better strength as well as a taller stature, especially in sprint races
- Females can close the gap to males in cold water over longer distances because of the insulating effect and better buoyancy in the water of their generally higher body fat percentage.
- Freestyle swimming is the fastest swimming stroke in ice swimming, and colder water temperatures do not always result in slower race times as psychological and physical cold habituation might minimize the effects of low temperatures on the body.

## Introduction

Winter swimming, also known as ice swimming or cold-water swimming, has developed from a national tradition in Nordic and Eastern countries of Europe such as Finland, Russia, and the Baltic states into an internationally known and health-improving sport [1]. Ice swimming has been proven to benefit the cardiovascular, immune, and endocrine systems and psyche [2]. Beginning as a personal challenge of endurance and the spirit, it has evolved into an internationally recognized sport with several organizational bodies complete with rules, regulations and record-keeping [2].

Nowadays, ice or cold-water swimming is considered a health-improving sport and is also carried out in international competitions [1, 3]. In 2009 Ram Barkai, the first man who completed an official “Ice Mile,” founded the International Ice Swimming Association (IISA), intending to introduce ice swimming as an internationally recognized sport [3, 4]. In the meantime, the IISA is organizing worldwide competitions in ice swimming and pursuing the dream of integrating ice swimming into the Winter Olympic Games by 2022 [5]. The International Winter Swimming Association (IWSA), founded in 2016, is another big association organizing and supervising global winter swimming competitions in open water [1]. In the events of the IWSA, female and male athletes from all over the world can participate in various swimming competitions. Three different swimming strokes, head-up breaststroke, butterfly, and freestyle, are offered. Head-up breaststroke and freestyle performance for races with distances of 25 m, 50 m, 100 m, 200 m, 450 m and 1000 m and the butterfly stroke over a distance of 25 m. Depending on the location and season of the event, the water temperature can range from − 2 to + 9 °C [1]. Water temperatures between − 2 and 2 °C are classified as ice water, between 2.1 and 5 °C as freezing water, and between 5.1 and 9 °C as cold water [1].

In recent years, the difference in performance between women and men in various sporting disciplines was examined in more detail [6–10]. Previous work showed an increase in the participation of women in elite sports disciplines and an improvement in the sex-related performance (8–12%) [11]. Men present physiological advantages, including larger body size, more skeletal muscles, higher muscle strength and power, lower body fat, and greater maximal anaerobic and aerobic energy delivery. Especially in the upper part of the body, men have about 36% more muscle mass, resulting in a larger sex difference than average in events where upper-body power is essential (i.e., canoeing with a sex difference > 20%) [12]. Sex differences in leg- or whole-body exercise sports in triathlon performances were analyzed, reporting swimming to be less affected by sex than cycling and running [6]. In ultra-marathon running, women have reduced the performance gap with advancing age [7]. Tower running is another discipline where women can outperform men but only in specific situations [8].

In swimming, research has focused mainly on long-distance and open water swimming. This discipline is known for a smaller sex difference than other sporting disciplines. It is a sport where certain aspects of women's body composition, like better hydrodynamic properties and floating skills, a lower drag coefficient, and a higher percentage of body fat, may give an advantage in performing at long durations, especially in cold water [6, 13]. Interestingly, women can outperform men in water colder than + 20 °C during solo, long-distance swimming events [9]. Girls at a young age (i.e., younger than ten years) and women in older age groups (i.e., above 80 years) can perform at the same level as men in all pool swimming disciplines [9]. Looking at the performance of open-water ultra-swimming, crossing the “English Channel,” over the last 36 years, the top three male swimmers were about 12% faster than their top female competitors, although the water temperature was over 15 °C [10]. Even though some data about women's performance compared to men exist in disciplines like swimming, running, and cycling, no studies have focused on the performance differences in cold water or the performance differences concerning the swimming stroke used.

Earlier studies showed that men are faster over shorter distances than women in swimming, whereas the sex difference in swimming performance decreased with an increasing distance [14, 15]. Furthermore, research in pool swimming showed that the sex difference in freestyle swimming from 50 to 1500 m became progressively smaller with increasing race distance [16]. Colder water temperatures resulted in an advantage for women due to their higher body fat percentage than men [2, 9]. Furthermore, specific swimming strokes, especially freestyle performance, lead to a higher sex-related difference in swimming speed than butterfly and classic head-up breaststroke [17, 18]. The “International Winter Swimming Association” (IWSA) clearly defined and set out the distances and permitted swimming strokes for their events. Distances are shorter than in classic pool swimming events, starting with 25 m instead of 50 m and do not exceed the length of 1000 m. Most races are performed using either “head-up breaststroke” or freestyle, the fastest swimming stroke [18]. However, classic backstroke is not a currently permitted swimming stroke. Moreover, the butterfly stroke is only performed at a 25 m distance. All disciplines are the same for male and female participants, allowing for sex-specific comparisons.

Accordingly, the present study investigates the sex difference in performance in women and men winter swimmers for different strokes on a 25 m and 200 m distance in the multiple stages of the Winter Swimming World Cup since 2016. Based on recent findings for the discipline of pool as well as cold-water swimming, we hypothesized that the gap in performance of sexes in winter swimming in the water below 9 °C would decrease with increasing swimming distance, in connection with lower water temperatures and that this decrease would be dependent on the swimming stroke used.

## Materials and Methods

### Ethical Approval

The study was conducted according to the guidelines of the Declaration of Helsinki. This study was approved by the Institutional Review Board of Kanton St. Gallen, Switzerland, with a waiver of the requirement for informed consent of the participants as the study involved the analysis of publicly available data (EKSG 01-06-2010).

### Data Source

Data included in this study was obtained from the “International Winter Swimming Association” (IWSA) [1]. The IWSA publishes all official results of ice swimmers competing in the multiple stages of the Winter Swimming World Cup on their website [1]. The results include the swimmer's full name, sex, age group, nationality, distance, type of stroke, and the completion time of each event. All available results of female and male ice swimmers from season 2016/2017 to season 2019/2020 have been included. The last event in 2020, “2nd Open Winter Swimming Championships of Karelia,” and all IWSA World Cup events in season 2020/2021 have been canceled due to travel limitations for international swimmers associated with the pandemic situation. The ambient temperature, humidity and barometric pressure were taken from historical weather reports [19]. Data included in the present study are presented in Table 1.

**Table 1:** Data included

Season	Event	City	Data included	Water temperature	Ambient temperature (°C)	Humidity (%)	Barometric pressure ( hPa)
2015/2016	7th Jelgavas Roni Cup	Jelgava	Excluded*	–	5.13	87	1019.79
2015/2016	4th Big Chill Swim	Windermere	Excluded*	–	6.36	95	988.00
2015/2016	5th Scandinavian Winter Swimming Championships	Skellefteå	Excluded*	–	− 2	100	992.21
2015/2016	7th Piritia Open	Tallinn	Excluded*	–	− 1.35	86	1004.22
2015/2016	10th Winter Swimming World Championships 2016	Tyumen	Excluded*	–	− 4	59	1018.99
2016/2017	8th Jelgavas Roni Cup	Jelgava	Included	+ 2.1 up to + 5	− 0.58	100	1012.80
2016/2017	1st Russian Pacific Winter Swimming Festival	Vladivostok	Included	+ 2.1 up to + 5	4	93	1014.43
2016/2017	5th Big Chill Swim	Windermere	Included	+ 5.1 up to + 9	8.75	97	1016.70
2016/2017	3rd Taierzhuang International Winter Swimming Festival	Taierzhuang	Included	+ 5,1 up to + 9	3	57	1024.00
2016/2017	6th Scandinavian Winter Swimming Championships	Skellefteå	Included	− 2 up to + 2	− 10° C	86	1032.97
2016/2017	8th Pirita Open	Tallinn	Included	− 2 up to + 2	− 0.31	87	1001.24
2017/2018	9th Jelgavas Roni Cup	Jelgava	Included	+ 2.1 up to + 5	5.88	93	1007.80
2017/2018	2nd Russian Pacific Winter Swimming Open Cup	Vladivostok	Included	+ 2.1 up to + 5	− 5	51	1018.43
2017/2018	3rd Minsk Open Cup	Minsk	Included	− 2 up to + 2	− 5.29	100	974.36
2017/2018	7th Scandinavian World Championships 2018	Skellefteå	Included	− 2 up to + 2	− 7	100	1002.15
2017/2018	11th Winter Swimming World Championships 2018	Tallinn	Included	− 2 up to + 2	− 5.92	64	1001.24
2018/2019	10th Jelgavas Roni Cup	Jelgava	Included	+ 5.1 up to + 9	11.46	88	1018.79
2018/2019	Bled Winter Swimming World Cup 2019	Bled	Included	+ 5.1 up to + 9	− 1.92	87	957.19
2018/2019	Skellefteå Dark& Cold 2019	Skellefteå	Included	− 2 up to + 2	6	53	988.24
2018/2019	Petrozavodsk Russian Open Championships 2019	Petrozavodsk	Included	− 2 up to + 2	4	56	1011.46
2019/2020	11th Jelgavas Roni Cup	Jelgava	Included	+ 5.1 up to + 9	3.69	75	999.81
2019/2020	5th Tyumen Open Cup	Tyumen	Included	+ 2.1 up to + 5	− 9	86	985.45
2019/2020	8th Winter Spring- Swimming (Daming Lake) International Invitational	Jinan	Included	+ 2.1 up to + 5	3	100	1016.37
2019/2020	12th Winter Swimming World Championships	Bled	Included	+ 5.1 up to + 9	− 1.35	45	981.07
2019/2020	9th Scandinavian Winter Swimming Championship	Skellefteå	Included	− 2 up to + 2	− 7	86	1002.15
2019/2020	2nd Open Winter Swimming Championships of Karelia	Karelia	Canceled	–	7	53	1009.47

All official results of the Winter Swimming World Cup from 2016 to 2020 were obtained from the IWSA website. *Canceled, no results available

#### Ice Swimming Events

The IWSA World Cup events feature three different swimming strokes: butterfly, head-up breaststroke, and freestyle. The butterfly stroke is only provided for 25 m races. Head-up breaststroke is a variant of the classic breaststroke typically performed in open water. Swimming breaststroke with the head above the water allows the swimmers to breathe without constraints and gives them a better orientation. Especially in ice water, the initial cold shock response and the likelihood of brain blood flow disruption, resulting in dizziness and increasing the risk of becoming unconscious and consequently drowning, is reduced [20]. Athletes can participate in 25 m, 50 m, 100 m and 200 m races for this swimming stroke. Swimmers who want to perform freestyle can participate in races of distances including 25 m, 50 m, 100 m, 200 m, 450 m and 1000 m.

#### Water Temperature Categories

The water temperature is clearly defined into event categories A, B and C by the International Winter Swimming Association for the IWSA World Cup [1]. Water temperature category A is classified as ice water (IW), ranging from a water temperature in Celsius of − 2° up to + 2° (including). Category B is defined as freezing water (FW), where temperatures lie between + 2.1° up to + 5° (including) Celsius. The third water temperature category, category C, is described as cold water (CW), where events occur at a water temperature between + 5.1° up to + 9° (including) Celsius. This classification of water temperature was considered for every race in this present study. The same applies to the age groups of the participating swimmers.

#### Age Group Categories

The IWSA introduced an age classification of 13 different groups, named from A1 to J2. Category A1 includes all swimmers under 15 years of age; A2 defines the age from 15 to 19 years of age. Category B (20–29) and C (30–39) have an age range of 10 years, whereas age category D (40–44), E (45–49), F (50–54), G (55–59), H (60–64), I (65–69), J (70–74) and J1 (75–79) have 5-year intervals. The last category, J2, is defined for 80 years and older. Those classifications are defined in the rules of the IWSA and are published on their homepage [1].

### Statistical Analysis

Descriptive data were presented by mean, standard deviation, maximum and minimum values or confidence intervals. For descriptive purposes, the nationalities were divided into six groups. Five nationalities were represented in the top 10 times in every season in all swimming strokes and water categories. According to Shapiro–Wilk and Levene's test, data did not follow a normal distribution nor had homogeneous variances. The Mann–Whitney *U* test was used to compare race time between swimming strokes of swimming (butterfly, freestyle or head-up breaststroke) and categories of water (IW, FW or CW). In addition, the Kruskal–Wallis *H* test was used to compare differences between water categories for the same sex; multiple strokes pairwise comparisons adjusted by Bonferroni correction were performed to identify the effect size of the differences between sexes. The level of significance set at 0.05. SPSS version 26.0 (SPSS, Inc., Chicago, IL, USA) was used for all statistical analyses.

## Results

### 25 m Events

A total of 6477 swimmers (2676 female and 3801 male), competed between 2016 and 2020 in 25 m races. Most of the swimmers (46%) competed in the IW category (*n* = 2980, 1305 female and 1675 male), followed by the FW (32.4%, *n* = 2099, 767 female and 1332 male) and CW (21.6%, *n* = 1398, 604 female and 794 male) category.

Considering the entire sample, the butterfly stroke (*n* = 1513) in the IW category had an overall mean time of 00:19.95 ± 00:00.28 (minimum 00:19.39/maximum 00:20.50) mm:ss.ms, in the FW category had a mean time of 00:21.63 ± 00:00.29 (00:20.59/00:21.74) mm:ss.ms and in the CW category had a mean time of 00:20.52 ± 00:00.36 (00:19.82/00:21.23) mm:ss.ms. Freestyle (*n* = 1881) presented in IW category a mean time of 00:19.76 ± 00:00.24 (00:19.30/00:20.23) mm:ss.ms, in the FW category a mean time of 00:20.25 ± 00:00.28 (00:19.71/00:20.79) mm:ss.ms and in the CW category a mean time of 00:19.26 ± 00:00.33 (00:18.62/00:19.90) mm:ss.ms. In the most used swimming stroke head-up breaststroke (*n* = 3083) in IW races had a mean time of 00:25.57 ± 00:00.17 (00:25.24/00:25.90) mm:ss.ms, in the FW category had a mean time of 00:26.35 ± 00:00.23 (00:25.90/00:26.79) mm:ss.ms and in the CW category had a mean time of 00:25.87 ± 00:00.27 (00:25.35/00:26.40) mm:ss.ms. The mean time spend in each stroke and water temperature category by each sex are given in Table 2.

**Table 2:** Meantime in each stroke and water temperature category by sex in 25 m races

Stroke	Water category	Sex	n	Mean time	SD		Minimum	Maximum	*p* Value	Power	Effect size
Butterfly	Cold water	Female	140	00:22.6	±	00:07.0	00:56.8*	00:15.1	< 0.001	0.691	− 0.40
		Male	220	00:18.5	±	00:04.9	00:39.4	00:12.0			
	Freezing water	Female	194	00:23.3	±	00:07.4	01:05.5*	00:14.0	< 0.001	0.774	− 0.35
		Male	372	00:19.0	±	00:05.9	00:55.7	00:12.2			
	Ice water	Female	221	00:21.2	±	00:05.9	00:48.7*	00:13.8	< 0.001	0.714	− 0.32
		Male	366	00:18.7	±	00:06.3	01:15.6	00:11.6			
Freestyle	Cold water	Female	183	00:21.4	±	00:07.2	00:54.7*	00:14.0	< 0.001	0.858	− 0.42
		Male	241	00:17.1	±	00:04.5	00:37.1	00:12.0			
	Freezing water	Female	224	00:22.5	±	00:07.2	00:49.0*	00:13.2	< .001	.814	− 0.34
		Male	411	00:18.0	±	00:05.2	00:45.6	00:11.2			
	Ice water	Female	326	00:22.4	±	00:08.4	01:03.9*	00:13.1	< 0.001	0.987	− 0.39
		Male	496	00:17.0	±	00:04.5	00:46.2	00:11.0			
Head-up breaststroke	Cold water	Female	281	00:28.7	±	00:07.8	01:13.7*	00:16.7	< 0.001	0.990	− 0.45
		Male	333	00:23.0	±	00:06.1	00:55.6	00:14.9			
	Freezing water	Female	349	00:29.4	±	00:08.8	01:21.3*	00:13.3	< 0.001	0.990	− 0.42
		Male	549	00:23.3	±	00:06.9	00:57.2	00:14.4			
	Ice water	Female	758	00:28.6	±	00:08.0	01:08.5*	00:15.0	< 0.001	0.999	− 0.43
		Male	813	00:22.6	±	00:05.8	00:50.1	00:13.4			

Data expressed in (mm: ss. ms); *p < 0.05 (sex difference for the same swimming stroke and water category)

**Fig. 1:**
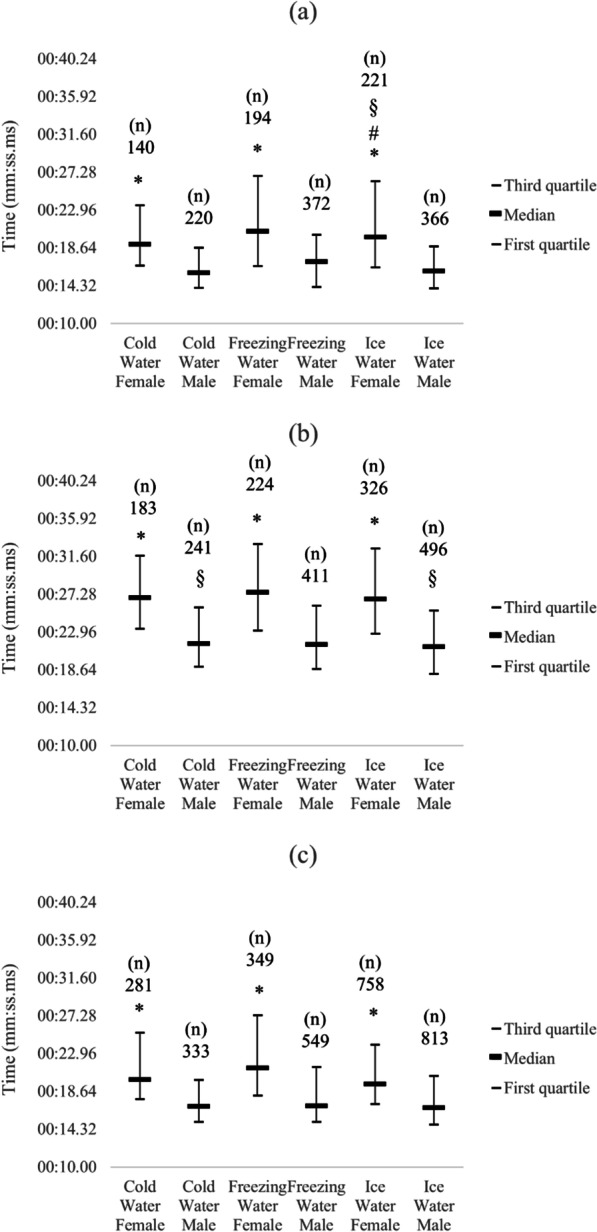
Meantime in each swimming stroke by water category for both sexes in 25 m races. *Different between sexes (*p* < 0.05), # different from the same sex in Cold water (*p* < 0.05), § different from the same sex in Freezing water (*p* < 0.05), **a** Butterfly, **b** Freestyle, **c** Head-up Breaststroke

Sex differences were observed between all the swimming strokes for all water categories, and effect sizes of the sex differences range from 0.34 to 0.45 (Fig. 1). Comparison among different strokes (25 m) for each water category and sexes showed interesting results. Head-up breaststroke was the slowest stroke (slower than freestyle and butterfly) for both sexes and water categories (*p* < 0.05). The butterfly stroke was slower than freestyle stroke for all water categories among the male athletes (*p* < 0.05). On the other hand, for females, butterfly was no different from freestyle stroke for ice (*p* > 0.05) and freezing waters (*p* > 0.05); however, it is slower than freestyle for cold water (*p* < 0.05).

### 200 m Events

A total of 1089 swimmers (408 female and 681 male) competed between 2016 and 2020 in 200 m races. Most of the swimmers (44.4%) competed in the IW category (*n* = 483, 167 female and 316 male), followed by CW category (34.5%, *n* = 376, 170 female and 206 male) and FW (21.1%, *n* = 230, 71 female and 159 male).

Considering the entire sample, most competed in swimming stroke freestyle (*n* = 724) in IW category had an overall meantime of 03:33.31 ± 01:04.53 (01:56.14/07:34.48) mm:ss.ms, in FW category had a mean time of 03:34.48 ± 01:06.97 (02:02.60/09:38.60) mm:ss.ms and in CW category had a mean time of 03:14.78 ± 00:48.51 (01:59.49/06:32.85) mm:ss.ms. In head-up breaststroke (*n* = 365) (there were 3083 swimmers in head-up breaststroke over 25 m but just 365 over 200 m) IW races had a mean time of 04:09.19 ± 01:01.80 (00:25.24/00:25.90) mm:ss.ms, FW category had a mean time of 04:15.98 ± 01:05.42 (02:52.46/07:55.71) mm:ss.ms and CW category had a mean time of 04:00.55 ± 00:49.55 (01:44.88/06:12.15) mm:ss.ms. The mean time spent in each stroke and water category by each sex are given in Table 3.

**Table 3:** Meantime for each swimming stroke and water category by sex in 200 m races

Stroke	Water category	Sex	n	Mean time	SD		Minimum	Maximum	*p* Value	Power	Effect size
Freestyle	Cold water	Female	95	03:33.0	±	00:53.3	02:16.2	06:32.9*	< 0.001	0.266	− 0.36
		Male	107	02:58.6	±	00:37.1	01:59.5	05:17.4			
	Freezing water	Female	51	03:53.0	±	01:05.3	02:19.1	07:02.9*	0.004	0.083	− 0.22
		Male	118	03:26.5	±	01:06.3	02:02.6	09:38.6			
	Ice water	Female	124	03:55.0	±	01:13.4	02:18.1	07:34.5*	< 0.001	0.158	− 0.24
		Male	229	03:21.5	±	00:55.9	01:56.1	07:14.6			
Head-up breaststroke	Cold water	Female	75	04:19.5	±	00:47.7	02:53.7	06:12.1*	< 0.001	0.171	− 0.34
		Male	99	03:46.2	±	00:46.2	01:44.9	05:40.9			
	Freezing water	Female	20	04:58.5	±	01:24.4	03:18.8	07:55.7*	0.004	0.080	− 0.36
		Male	41	03:55.2	±	00:41.1	02:52.5	05:39.5			
	Ice water	Female	43	04:22.6	±	01:01.6	02:24.1	07:50.0*	0.010	0.128	− 0.23
		Male	87	04:02.5	±	01:01.2	02:48.2	08:40.3			

Data expressed as (mm:ss.ms); *p < 0.05 (sex difference for the same swimming stroke and water category

**Fig. 2:**
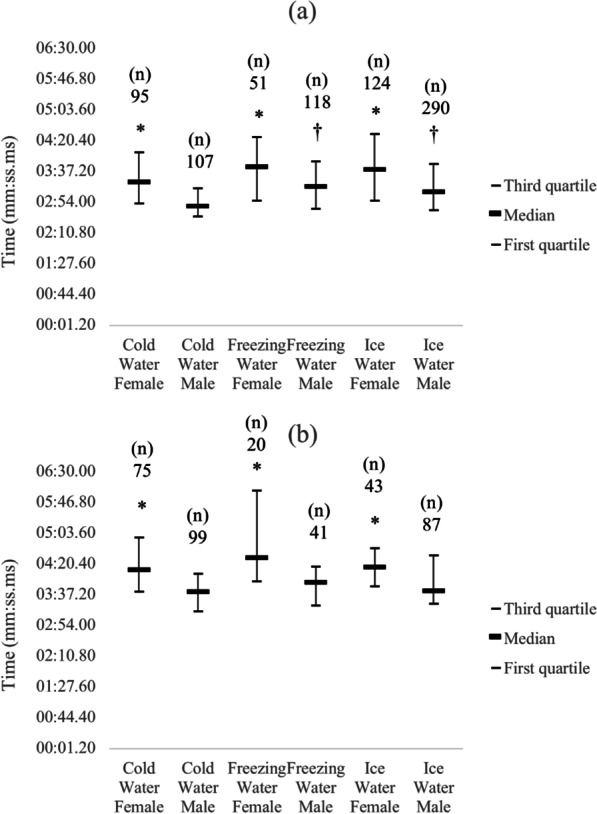
Meantime for each swimming stroke by water category for both sexes in 200 m races. *Different between sexes (*p* < 0.05), † different from Cold Water for the same sex (*p* < 0.05), **a** Freestyle, **b** Head-up Breaststroke

Sex differences were observed between all the swimming strokes for all water categories, and effect sizes of the sex differences range from 0.22 to 0.36 (Fig. 2). Differences among water categories for each stroke and sex also are shown in Fig. 2. Comparison between 200 m freestyle and head-up breaststroke showed that the freestyle was a faster stroke for all the water categories and sexes (*p* < 0.05).

## Discussion

The present study investigated the sex difference in swimming performance of female and male winter swimmers competing in the multiple stages of the Winter Swimming World Cup since 2016. We hypothesized that the sex gap in performance of winter swimming in water below 9 °C would decrease with an increasing swimming distance in combination with lower water temperatures and that this decrease would be dependent on the swimming stroke used. The main results were that female athlete presented significantly longer race times for all strokes and water categories than male swimmers in 25 m and 200 m events. The difference between the sexes was greater in the 25 m than in the 200 m for all strokes and water temperatures. Female athletes presented longer race times in FW or CW than in IW for butterfly stroke, while male athletes presented no difference among water categories in 25 m events. For freestyle stroke, male athletes presented longer race times in FW than CW or IW, while female athletes presented no difference among water categories. Male and female athletes presented no difference in race time among water categories for head-up breaststroke.

### Influence of Sex on Different Race Distances

A part of our hypothesis was that women would decrease the performance gap in winter swimming over longer distances. The study shows that male athletes were significantly faster than female swimmers in butterfly, freestyle, and head-up breaststroke races over 25 m across the three water temperature (CW, FW, IW) categories. The finding that men were faster than women in sprint distances is most probably due to the differences in anthropometric characteristics such as body height [21], body composition [22] (e.g*., *body mass, body mass index, fat mass, body fat percentage, visceral adipose tissue level, muscle mass, total body water), muscle thickness [23] and muscle size [24] of men compared to women. In general, women's body composition showed lower values in the body mass index, fat mass per kg, muscle mass, visceral adipose tissue level but a higher body fat percentage than men. Men may outperform women due to the larger muscle mass and force production ability, resulting in a more significant stroke force in the water [25]. These muscular enhancements result in a rightward shift of the force–velocity curve, leading to faster finishing times [26].

The energy cost to move forward increases with speed, whether on land or water. However, in terrestrial sports, the energy cost is lower than in water since resistive forces in water (hydrodynamic resistance, drag, lower propelling efficiency) are larger than the aerodynamic force on land and need to be overcome. The determinants of the energy costs, like drag and efficiency, as well as energy expenditure in its aerobic and anaerobic components, play a role in the performance of athletes [27]. In sprint races, energy is produced mainly on the anaerobic system compared to endurance events, where the resynthesis of ATP relies more on the aerobic system (65%) than on the anaerobic (35%) [12, 28]. Sex differences for the aerobic system are recorded to be smaller than for anaerobic.

Although muscle mass, anaerobic power and force production are significant predictors of explosive and sprint ability in swimmers [26, 29–31], anthropometric characteristics (e.g., body height, body weight, body fat percentage, aerobic capacity) become an important contributor with increasing race distance [21, 32, 33]. The average height for Olympic swimmers in 2016 was 188 cm for men and 175 cm for women [34]. While not measured in the current study, male swimmers tend to be taller than their female competitors. Differences in average height are often associated with higher muscle mass, resulting in longer limb levers and, therefore, a more potent stroking force and faster performance on a sprint distance in swimming [21–25, 34].

Secondly, previous evidence suggested that in longer distances in cold water temperatures, such as the 200 m, we were expecting that women’s higher level of body fat, giving them a better buoyancy and insulation [35, 36], would have a positive effect on their race time compared to men [2, 9, 37]. The present study revealed that the effect size of the sex differences on 200 m was smaller than on 25 m for all strokes and water temperatures, supporting our hypothesis. The assumption was based on scientific research showing female swimmers recorded thicker skinfold scores and, therefore, more subcutaneous fat. This causes women to retain more heat within the body for a longer duration and delay muscle cooling to a fatiguing level so that they may retain their swim speed for a longer time in cold water than males with less body fat [38]. A study investigating the body composition in female and male open water swimmers during a competition in Zurich Lake revealed that women have around 12% more body fat than men, which gives them a better basis in cold water over longer distances [39]. Besides that there is evidence that women have different metabolic and hormonal responses to cold water immersion than men [40]. However, a significant difference in the thermogenic response could not be detected. Cold acclimation showed to increase the brown adipose tissue activity and non-shivering thermogenesis [41]. Thus, one can postulate that women with a larger body fat percentage would benefit from that thermogenesis more than men. Other sources report superior cold tolerance of women compared to men when resting in cold water [42]. Previous experience seems to be an important factor apart from anthropometric characteristics for success in ice swimming, especially over longer distances [43].

Furthermore, research showed that psychological skills training could help swimmers suppress the drive to breathe during cold water immersion [44]. However, in this study comparing the performance of female and male athletes on a sprint distance of 25 m to a middle distance of 200 m, the length of the middle-distance track was relatively short. The positive effect of women’s higher level of body fat, which gives them better insulation in cold water and induces a trunk incline giving the body a more streamlined and efficient position as well as better body buoyancy, might not have developed fully over a distance of 200 m [45]. Besides that, time in water over a distance of 50 m and 200 m might be too short since body core temperature during ice swimming first increases and might not have dropped seriously [16, 43]. Comparing the present study results with elite swimming competitions in average temperatures, for example, at the Olympic Games 2020 in Tokyo, female and male Olympic participants only need half of the time for the same track [46]. Also, sex differences in ice swimmers for 1 km Ice Event were higher than pool swimmers [16]. Future studies need to investigate the performance of men and women on a sprint distance to longer distances (i.e., 450 m, 1000 m). Furthermore, the body composition, core temperature, hormonal activity and especially the fat mass of female and male ice swimmers competing at the international level should be scientifically investigated.

### Sex Differences in Swimming Speed for Butterfly Stroke in Relation to Water Category

An interesting finding was that women could not close the sex gap over 25 m butterfly stroke. This is possibly due to the muscular and energetic demands of the butterfly stroke. Compared to freestyle and backstroke, butterfly stroke has a significantly increased energy cost and anaerobic contribution [47]. Consequently, success in sprint butterfly is predicated on an anaerobic capacity determined by body composition [47, 48], specifically muscle mass and net force production ability. As a result, men are likely able to outperform women in such a short event requiring maximal force production.

It is important to note that men are able to produce faster completion times in butterfly stroke over 25 m; it is not possible to compare over longer butterfly distances. Currently, the IWSA only offers the 25 m event distance. Interestingly, the butterfly event has the lowest participation rate compared to freestyle and head-up breaststroke races over 25 m. The number of women competing in this event was considerably lower than the number of men (555 women and 958 men). Therefore, it is possible that this difference may contribute to this significant difference in performance. However, as an additional result of our study, we found that women’s butterfly swimming speed was faster in IW (− 2 °C to + 2 °C) than in FW or CW (over + 2°–9 °C) and closest to the performance of men with a mean time difference of 2.55 s. Regardless of gender, swimmers may exhibit a cold shock response, but the rate of muscle cooling would be reflected in the insulation from subcutaneous fat. Therefore, men's times may slow to a greater extent than women's in IW. In contrast to women, men presented no differences in butterfly swimming speed for the different water categories over the sprint distance of 25 m. Further studies might investigate the sex difference in butterfly swimming over longer distances to reveal whether an improvement in women's performance can be observed over a longer distance in this discipline as well, especially in IW, where the performance gap appeared to be smallest. Besides that, it would be of great interest to examine different characteristics of the swimmers in terms of age and training status.

### Sex Differences in Swimming Speed for Freestyle Stroke in Relation to Water Category

We can confirm our hypothesis that the sex gap in water below 9 °C would decrease with an increasing swimming distance since the effect size of the difference between the sexes was greater in the 25 m race than in the 200 m race for all water categories when swimming freestyle. Women's performance was most similar to men during the swim on a middle distance of 200 m in FW with an effect size of 0.22. Interestingly, freestyle is faster than butterfly stroke over 25 m for female athletes only in the cold and freezing water category. In addition, freestyle is the most popular stroke used by swimmers during training [49]. In contrast to freestyle, the breaststroke is a less popular stroke; those with hip and knee problems may struggle to breaststroke without pain [50]. This may explain why more swimmers take part in the 200 m freestyle events (*n* = 724) of the IWSA World Cup events than in the 200 m head-up breaststroke events (*n* = 365). Future studies might compare the best female to the best male athletes in this discipline to find the true sex difference. Furthermore, the race times of winter swimmers in the morning and afternoon might be compared since the time of practice is an essential factor in the economy of movement. Studies showed that Olympic swimmers were faster in the late afternoon than in the morning [51].

Secondly, the result of the present study confirms that freestyle is a faster stroke than butterfly or head-up breaststroke for male athletes across all water categories. Interestingly, women's performance during freestyle swimming in relation to the water categories' speed remained unchanged, although men’s freestyle swimming speed over 25 m and 200 m was the slowest in FW (+ 2.1 °C to + 5 °C). Despite adequate preparation and conditioning, rapid changes in environmental conditions (i.e., cold water immersion) have been shown to affect neuromuscular and musculoskeletal systems [2, 52, 53]. Women may have an anthropometric advantage due to shorter stature and a higher body fat percentage, which confers more excellent cold water resistance compared to their male counterparts.

### Sex Differences in Swimming Speed for Head-Up Breaststroke in Relation to Water Category

Furthermore, we can confirm our hypothesis that the sex gap in water below 9 °C would decrease with an increasing swimming distance, also for the head-up breaststroke. Men were faster than female swimmers in all water categories, but the effect size of the difference between sexes was greater in the 25 m, ranging from 0.43 to 0.45 than in the 200 m where the effect size between sexes ranges from 0.23 to 0.36. Especially in the IW category, it is noticeable that female swimmers reduced the effect size of sex differences from 0.43 in 25 m races to 0.23 in 200 m races. Generally, head-up breaststroke swimmers' highest tethered swimming force values are recorded [25, 54, 55]. This can be explained by the powerful leg kick of this stroke compared to other swim strokes where the leg's action is primarily to keep body balance [54]. However, the head-up breaststroke appears to be the slowest stroke compared to other swimming strokes [56]. The present study confirms that head-up breaststroke is the slowest stroke for men and women in all water categories. This might be the over-water recovery of the arms and lower continuity of the synchronization of arms and legs. Moreover, female and male athletes presented no difference in race time among the water categories. Future studies should compare the body fat of women and men competing in IW to determine if the smallest difference between sexes in IW is related to body composition.

### Limitations and Implications for Future Research

A limitation of this study is the short time frame of four years and the low number of female athletes. A comparatively lower number of swimmers participated in longer events like 450 m and 200 m than in the 25 m. We assume that the sex difference will be even lower with a higher number of successful women. Further studies should include longer distances (i.e., 450 m, 1000 m) to confirm the decreasing sex difference with an increasing distance. Furthermore, this study is exclusively retrospective and descriptive. Potential important variables such as body weight, body height, previous experience, training status, motivation and weather conditions were not considered.

## Conclusions

In ice swimming in water temperatures below 9 °C, male athletes were generally faster than females for all strokes and water categories in the 25 m and 200 m events of the IWSA World Cup, but women were closing the gap at the longer distance. The effect size of the sex differences on 200 m (0.22–0.36) was smaller than on 25 m (0.34–0.45) for all strokes and water temperatures. Colder water temperatures do not always mean slower times; however, there were differences related to stroke.

## Data Availability

The swimmers' data were downloaded from the official IWSA website (https://iwsa.world).

## Abbreviations

CW: Water category C; Cold Water + 5.1° up to + 9° of Celsius
FW: Water category B; Freezing Water + 2.1° up to + 5° of Celsius
IISA: International Ice Swimming Association
IW: Water category A; Ice Water − 2° up to + 2° of Celsius
IWSA: International Winter Swimming Association
